# Uptake of Australia’s Health Star Rating System 2014–2019

**DOI:** 10.3390/nu12061791

**Published:** 2020-06-16

**Authors:** Maria Shahid, Bruce Neal, Alexandra Jones

**Affiliations:** 1 The George Institute for Global Health, UNSW, Sydney, NSW 2042, Australia; bneal@georgeinstitute.org.au (B.N.); ajones@georgeinstitute.org.au (A.J.); 2 Charles Perkins Centre, University of Sydney, Sydney, NSW 2006, Australia; 3 Department of Epidemiology and Biostatistics, School of Public Health, Faculty of Medicine, Imperial College London, London SW7 2AZ, UK

**Keywords:** front-of-pack, food labelling, health star rating, nutrient profiling

## Abstract

In June 2014, Australia and New Zealand adopted a voluntary front-of-pack nutrition label, the Health Star Rating (HSR) system. Our aim was to assess its uptake in Australia in the five years following adoption and examine the feasibility of proposed targets for future uptake. Numbers and proportions of products eligible to carry a HSR were recorded each year between 2014 and 2019 as part of an annual survey of four large Australian retail outlets. Uptake was projected to 2024. Mean HSR values were determined for products that were, and were not labelled with a HSR logo, and summary data presented overall, by HSR score, by major food category, by manufacturer and manufacturer group. Differences in mean HSR were assessed by independent samples *t*-test. HSR uptake continues to increase, appearing on 7118/17,477 (40.7%) of eligible products in 2019. Voluntary display of the HSR logo was increasing linearly at 6.8% annually. This would need to be maintained to reach 70% by 2024. Of those products displaying a HSR logo, more than three quarters (76.4%) had a HSR ≥ 3.0. Products displaying a HSR logo had a significantly higher mean HSR (3.4), compared to products not displaying a HSR logo (2.6) (*p* < 0.001). One hundred and thirty-nine manufacturers were using HSR, but retailers Coles, Woolworths and ALDI were together responsible for the majority of uptake (55.9%). Manufacturer members of the Australian Food and Grocery Council were responsible for 28.6% of uptake. Our findings illustrate the limits of commercial goodwill in applying HSR voluntarily. Ongoing implementation must pair clear targets and timelines for uptake with a firm pathway to make HSR mandatory if sufficient progress is not achieved.

## 1. Introduction

The World Health Organisation (WHO) recommends front-of-pack nutrition labelling (FoPL) on packaged foods and beverages as part of its suite of evidence-informed measures aimed at promoting healthier diets and preventing non communicable diseases (NCDs) [1,2]. FoPLs summarise the nutritional quality of a product to assist consumers in making healthier choices. The uptake of FoPLs continues to expand globally, with over 30 countries currently using some form of government-led FoPL system [3].

In 2014, Australia and New Zealand adopted the Health Star Rating (HSR) system as a voluntary FoPL, following a period of development led by federal, state and territory governments and involving consumer, industry and public health groups [4]. The HSR system summarises the nutritional quality of a product and assigns it a rating from 0.5 stars (least healthy) to 5.0 stars (most healthy) in ten half star increments. At its adoption, Food Ministers from both countries agreed to a formal five-year review (Review) of HSR to consider if, and how well, it was meeting its objectives and to identify options for improvements [4].

In 2019, a government-appointed Independent Reviewer delivered their recommendations on HSR in a Five Year Review Final Report [5]. Food Ministers subsequently issued a formal response, broadly supporting the Independent Reviewer’s findings [6]. Overall, the Review concluded that HSR was performing well and recommended that it be continued. It also recommended a package of reforms. These include refinements to HSR’s algorithm for scoring foods in response to multiple rounds of public consultation and modelling to address outliers and ‘anomalies’ in product scores, the limitations of which have been the subject of high profile media attention and peer-reviewed publications [5,6,7,8]. With respect to HSR’s graphic design, improvements include removal of the non-interpretive ‘energy icon only’ variant of the HSR label (Appendix A—Figure A5) as an option for manufacturers to use on pack. Other recommendations that will be incorporated into reforms include improvements to HSR’s governance to increase government oversight in HSR’s management and monitoring, including a greater role for Food Standards Australia New Zealand (FSANZ). The role of HSR’s existing governance committees and their membership will also be clarified. While the government remains HSR’s ultimate decision-maker and funder, day-to-day implementation has been guided by the multi-stakeholder Health Star Rating Advisory Committee (HSRAC), which includes food industry representation despite public health and consumer stakeholders raising concerns about their potential commercial conflicts of interests [9,10].

A major focus of the Review was whether HSR should be made mandatory. On its introduction, Food Ministers agreed that HSR could remain voluntary subject to ‘consistent and widespread uptake.’ At that time they suggested that if voluntary implementation was found unsuccessful, a mandatory approach would be required [11], though no targets or performance indicators of satisfactory uptake were established. In 2017, three years after HSR’s introduction, our independent research found that HSR was on 28% of eligible products in Australia, skewed disproportionately towards products scoring higher HSR values [12]. Figures included in the Review Report suggest HSR was on 31% of eligible products in 2018, or 38% of household food purchases in Australia [5]. In New Zealand, only 21% of eligible products were displaying HSR [5]. While public health and consumer groups have supported making the system mandatory to provide consumers with HSR’s full benefit [13,14], peak food and beverage industry groups including those represented on the HSRAC continue to strongly oppose this move [14]. In considering these competing views, the Review ultimately concluded that HSR could remain voluntary while recommended improvements are made. At the same time, the Review recommended that if uptake did not reach 70% within a further five years, HSR should be mandated [5]. In their response, Food Ministers supported this recommendation subject to the development and agreement upon interim and final target metrics [6].

The aim of this analysis is to update progress in HSR uptake in Australia in 2019 and provide insight into the utility and feasibility of interim and final target metrics.

## 2. Methods

### 2.1. Data Source

The George Institute’s FoodSwitch program captures images of packaged foods and beverages using a bespoke mobile application, allowing for the extraction and collation of key food labelling and food composition data [15]. Using this process, the FoodSwitch Monitoring Datasets are generated annually based on systematic data collection from four large Australian supermarkets owned by Aldi, Coles, Independent Grocers of Australia (IGA) and Woolworths in the Sydney metropolitan area. In-store surveys of all foods and beverages in these stores are conducted by trained data personnel who capture images of key information including product barcode, product name, FoPLs, health and nutrient content claims, package size, ingredients list, manufacturer and brand names, and the Nutrition Information Panel (NIP). Data is entered into the Monitoring Database using these images by trained data entry personnel.

We used the FoodSwitch Monitoring Datasets for the years 2014, 2015, 2016, 2017, 2018 and 2019 to compare annual uptake of HSR on pack since the system was implemented.

### 2.2. Product Categorisation and Eligibility for HSR 

Using the categorisation system developed by the Global Food Monitoring Group, foods and beverages in the FoodSwitch program are classified into a hierarchical category tree to allow for comparison of nutritionally similar foods [16]. Products are categorised into major categories (e.g., ‘Bread and bakery products’), minor categories (e.g., biscuits, bread, etc.) and further levels of subcategories.

Our analysis included packaged food and beverage products, and excluded the following categories as they have been specifically deemed out of scope of the HSR system: alcoholic beverages; formulated supplementary sports foods; infant foods and formulas; meal kits; foods for special medical purposes; vitamins and supplements [17,18]. This left 15 major categories for analysis. Within this, we also excluded subcategories of baking sodas and powders; chewing gum; herbs and spices; plain teas and coffees; yeasts and gelatines, as these foods do not contribute significantly to nutrient intake, are not required to display a NIP [19], and also therefore not required to display a HSR. We further excluded variety packs as the FoodSwitch system is unable to generate a HSR for products with multiple NIPs.

Products were identified using their unique barcode. Where a product appeared in more than one package size (i.e., 375 mL can or 600 mL bottle of the same drink), each package size was counted as an individual product. This approach captures the number of product packages that have been updated by manufacturers to display HSR.

### 2.3. Food Labelling and Food Composition Data

**Figure A2:**
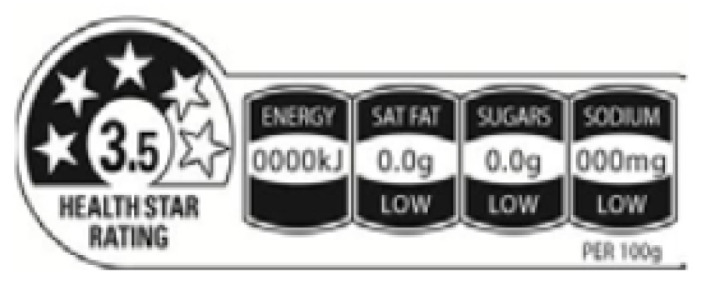
Health Star Rating + energy icon + 3 prescribed nutrient icons.

**Figure A3:**
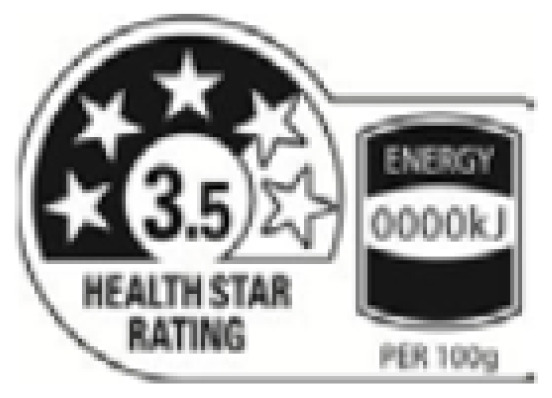
Health Star Rating + energy icon.

**Figure A4:**
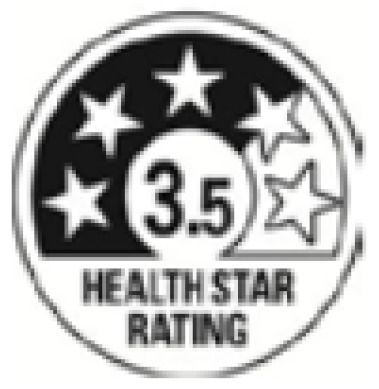
Health Star Rating only.

The presence or absence of HSR labelling has been routinely determined at data entry since 2015 by examining images of product labels. Where HSR labelling was being used, we recorded whether the label used the full HSR logo with star graphic (Appendix A—Figure A1, Figure A2, Figure A3, and Figure A4), or the energy icon only variant (Appendix A—Figure A5). Where a HSR logo was present, we recorded the HSR value displayed (from 0.5 to 5.0). The presence of HSR was not systematically recorded in 2014 as this was the year of HSR’s introduction and is therefore taken as zero for this paper.

For all products in the 2019 Monitoring Dataset, we also extracted information from the NIP on the back of pack. Energy (kJ/100 g), protein (g/100 g), saturated fat (g/100 g), total sugar (g/100 g), and sodium (mg/100 g) are mandatory on the Australian nutrient declaration but details on Fruit Vegetable Nut and Legume content (FVNL) (%), concentrated FVNL (%), and fibre (g/100 g) are optional. Where such details were not provided by the manufacturer on the package, appropriate levels were estimated using information drawn from the back-of-pack ingredients list, generic food composition databases, or by analogy with similar products using methods described previously [15]. The estimation process provides a proxy value for each nutritional indicator at the finest category level for more than 700 individual food subcategories. Proxy values are then substituted for each product in that category for which data are missing.

We also extracted the manufacturer of each product. Each manufacturer was identified by its Australian trading name. We grouped manufacturers into three mutually exclusive categories to further understand potential drivers of uptake: (1) grocery retailers making private-label products (Coles, Woolworths, ALDI and IGA); (2) non-retail manufacturers represented on the HSRAC; and (3) all other manufacturers. The two industry representatives on the HSRAC from 2014–2019 were the Australian Food and Grocery Council (AFGC) and the Australian Industry Group (AiGroup). Manufacturers were flagged as AFGC members using the list in the AFGC’s 2018–2019 Annual Report [20]. The AiGroup purportedly represents the confectionery sector, but does not disclose individual members publicly, and declined the authors’ invitation to provide this information on request [21]. At least some major confectionery manufacturers are represented by the AFGC, otherwise AiGroup members were captured in the broad group of ‘all other manufacturers.’

### 2.4. Calculation of HSR Values

Where a product was displaying the HSR logo on its label, we used the HSR value displayed by the manufacturer for the purposes of our analysis.

For products where the HSR logo was not being displayed (either because the manufacturer had not adopted HSR, or had elected to display the energy icon only variant), we calculated the HSR value using the algorithm described in the ‘Guide for Industry to the Health Star Rating Calculator’ [18]. In summary, products were categorised into one of six HSR categories: non-dairy beverages; dairy beverages; oils and spreads; cheese and processed cheese; all other dairy foods; all other non-dairy foods. Depending on the HSR category, baseline points were assigned to a product using its energy, saturated fat, total sugar and sodium (g/100 g) content and modifying points assigned using its FVNL%, concentrated FVNL%, protein and fibre (g/100 g) content where applicable. A HSR ‘score’ was determined by subtracting the modifying points from the baseline points, which was then converted into a HSR from 0.5 to 5.0 stars based upon a defined scoring matrix for each of the six categories. A higher HSR indicates a healthier product in that category.

### 2.5. Statistical Analyses

In order to obtain the percentage uptake of HSR in the primary analysis, HSR uptake was determined separately for each year by dividing the number of products carrying the HSR logo or energy icon variant by the total number of eligible products. Analysis was completed using data for 2014 to 2019. To determine the feasibility of the proposed target metric of 70% HSR uptake in the next five years, we projected HSR uptake linearly from 2019 to 2024. We did this using two scenarios: (1) HSR uptake continues linearly to 2024 from total uptake in 2019 and (2) HSR uptake continues linearly, but from a baseline that includes only use of the HSR star logo (e.g., uptake does not include use of the energy icon only variant).

Based upon the Monitoring Database 2019 extract, we also determined the proportions of products displaying HSR by each HSR value 0.5–5.0, by 15 major food categories, and by manufacturer. In each case the mean HSR of products displaying the HSR logo was compared against the mean of all products eligible to carry the HSR but not displaying a logo, either because they do not use HSR at all, or use the energy icon only. Differences in means were assessed for statistical significance using an independent samples *t*-test (*p* < 0.05).

Data manipulation and analyses were conducted in Stata/IC version 15.1, and figures and linear trends were generated in Microsoft Excel.

## 3. Results

### 3.1. HSR Uptake over Time

**Figure 1:**
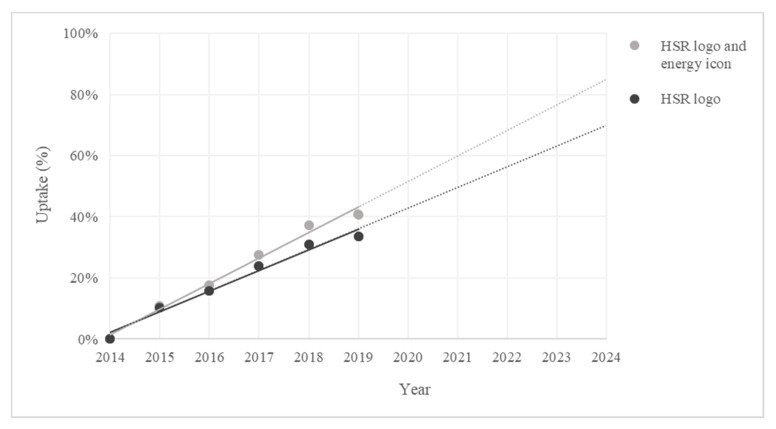
Australian uptake (%) of Health Star Rating to 2019 and projection to 2025. Linear trend for HSR system (HSR logo and energy icon) represented by the equation y = 0.0836x + 0.0142; R^2^ = 0.9869. Linear trend for HSR logo represented by the equation y = 0.0678x + 0.0212; R^2^ = 0.9787.

Within the FoodSwitch Monitoring Database in 2019 there were 7118 products using the HSR system out of 17,477 eligible products. Of these, 5858 (33.5%) were displaying the HSR logo, and 1260 (7.2%) the energy icon only. Together these products represented 40.7% of all HSR eligible products (Figure 1).

The trend for HSR uptake suggests an approximately linear increase of 8.4% per annum in products using any variant of HSR each year since the system was introduced. If this linear trend is maintained, uptake could reach approximately 85% by 2024. If use of the energy icon variant is removed from valid HSR uptake, use of the HSR logo has been increasing 6.8% per year, suggesting uptake could reach approximately 70% by the year 2024.

### 3.2. HSR Logo Uptake by HSR Value

**Figure 2:**
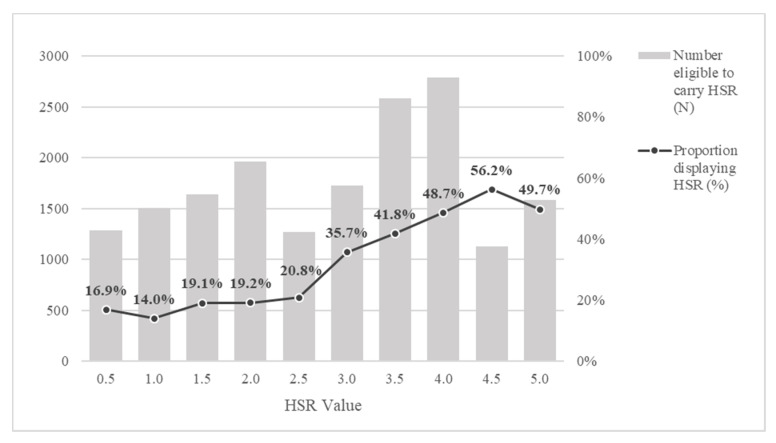
Uptake (%) of the HSR logo across HSR values in 2019.

Products receiving a higher HSR were more likely to use the HSR logo (Figure 2). Products scoring HSR 4.5 had the highest proportional uptake, with 56.2% of 1126 products eligible displaying the HSR logo. Conversely, the lowest uptake of the HSR logo was observed in products scoring HSR 1.0 (14.0%). Of the 5858 products displaying a HSR logo, 4475 (76.4%) displayed HSR ≥ 3.0.

### 3.3. HSR Uptake by Category

HSR uptake varied by category (Table 1). Categories with the highest uptake were ‘Fish and fish products’ (54.5%), ‘Fruit and vegetables’ (51.2%) and ‘Convenience foods’ (50.9%). Categories with the lowest uptake were ‘Sugars, honey and related products’ (19.1%), ‘Edible oils and oil emulsions’ (25.0%) and ‘Sauces, dressings, spreads and dips’ (28.1%).

In 12/15 categories the mean HSR of products displaying the HSR logo was significantly higher than the mean of those products not using the HSR system or displaying the energy icon only. This difference was greatest within ‘Non-alcoholic beverages’, where products displaying the HSR logo had a mean HSR 4.1 compared to a mean HSR 2.3 for products not displaying the logo (*p* < 0.001). 

The confectionery and non-alcoholic beverage categories were responsible for 685/1260 (54.4%) of products using the energy icon only. The majority of products (70.4%) using the energy icon would receive a HSR between 0.5–2.0.

### 3.4. HSR Uptake by Manufacturer and Manufacturer Group

Manufacturer uptake of HSR also varied. Table 2 provides individual results for manufacturers with ≥80 HSR eligible products. Manufacturers with the highest proportionate uptake across their portfolios were McCain (97.5%), Sanitarium (96.3%) and Coles (92.4%), though there was large variation in the number of products made by these manufacturers. 

Uptake by some larger manufacturers remained poor. Eight large manufacturers did not display HSR on any products in 2019: Mondelēz, Oriental Merchant, IGA, Manassen Foods, Parmalat, General Mills, Murray Goulburn Co-operative Company. The Market Grocer, Bega Cheese, Ricegrowers (SunRice), and San Remo Macaroni Company only displayed HSR on <10% of their products.

The mean HSR for products displaying a HSR logo on pack was significantly higher than the mean for products made by that manufacturer and not showing a HSR logo, except for the following manufacturers: McCain Foods, Coles, Woolworths, San Remo Macaroni Company and The Market Grocer. The greatest difference observed was for Nestlé (2.5 star difference; *p* < 0.001), Mars (2.2 star difference; *p* < 0.001), George Weston Foods (1.9 star difference; *p* < 0.001) and Bega Cheese (1.9 star difference; *p* = 0.008).

In total, 139 manufacturers were using HSR on at least one product.

**Figure 3:**
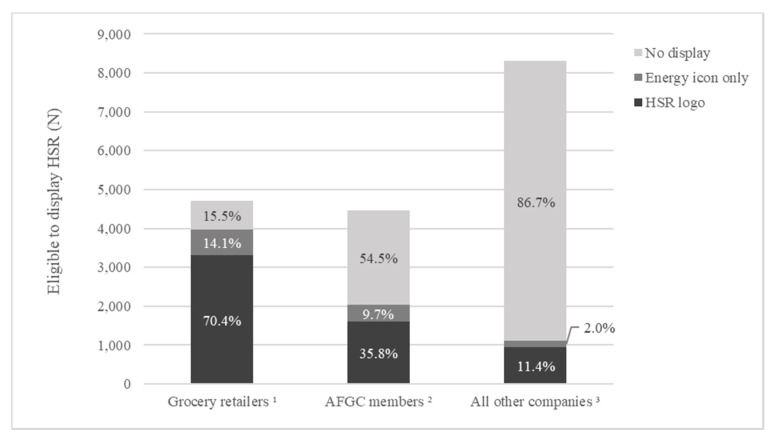
HSR uptake and type of HSR being displaying by company type. ^1^ Grocery retailers included: ALDI, Coles, Independent Grocers of Australia, Woolworths, ^2^ Australian Food and Grocery Council members included are as listed in the Annual Report [20], ^3^ 135 other companies display the HSR system on at least one of their products.

Together, grocery retailers Coles, Woolworths and ALDI were responsible for the majority of total HSR uptake in 2019 (n (%) = 3979 (55.9%)) (Figure 3). Of these, 3315 (70.4%) displayed the HSR logo and 664 (14.1%) displayed the energy icon only. The grocery retailer IGA did not display HSR on any of its products.

AFGC members together were responsible for 28.6% of total HSR uptake (2033 products). Of their eligible products, 1600 (35.8%) displayed the HSR logo, and 433 (9.7%) displayed the energy icon only. The majority of AFGC members’ products currently do not display the HSR system at all (n (%) = 2431 (54.5%)). It was not possible to assess uptake by AiGroup members.

All other manufacturers were responsible for 15.5% of total HSR uptake (1106 products). Most of these were using the HSR logo (n (%) = 943 (11.4%)).

## 4. Discussion

Uptake of HSR has increased steadily in Australia over its first five years of voluntary implementation. However, HSR is still only displayed on a minority of eligible products, mostly those that score well. Selective HSR use provides marketing benefit to manufacturers but restricts HSR’s utility as a public health intervention by denying consumers opportunity to make meaningful comparison between products and limiting their ability to identify and avoid less healthy foods.

Our findings update the latest government-issued estimates of HSR uptake included in the Review [5,6]. Those figures, resulting from government-commissioned Heart Foundation monitoring found that in 2018, 5448 products (or 30.5% of all eligible products) displayed HSR (logo and energy icon variants), and that sales weighted uptake was slightly higher, at 37.9% [22]. Trends in overall uptake and patterns of uneven HSR use are also consistent with our earlier 2017 findings, which showed that uptake of HSR was neither widespread nor consistent [12]. As recognised by Food Ministers in their own response to the Review, inconsistent uptake of HSR on products negatively affects consumer trust in the system, as well as reducing the actual effectiveness of HSR by allowing fewer opportunities for meaningful comparison between foods [6].

Our findings provide insights for government policymakers leading the next phase of HSR implementation. In this phase, the Review found that HSR should remain voluntary to allow attention to be focused on implementing agreed improvements, but with clear targets set and all stakeholders working together to drive uptake. Specifically, the Review also recommended that if HSR continues to perform well but is not displayed on 70% of products in a further five years, HSR should be mandated [6]. Our newly updated figures provide insight into the feasibility of this target. If a linear trend for uptake is maintained, 70% uptake is theoretically feasible by 2024, even if products currently using the now defunct energy icon are removed from consideration. This may provide a promising incentive for the industry to continue uptake. The publication of annual interim targets and transparent, regular monitoring against year-on-year progress would support accountability towards this goal [23]. However, even if this goal is attained after ten years of voluntary implementation, consumers would still miss the benefit of HSR on around 1/3 of eligible foods. Current trends in HSR usage suggest this remaining third are also likely to be unhealthy products at the lower end of the star rating spectrum.

There are also reasons to suggest continued linear uptake is unlikely. Our results suggest that grocery retailers, who account for the majority of uptake to date, have reached near-saturation in applying HSR to their large private-label product portfolios. This suggests continued uptake will require constructive and strategic engagement to obtain buy-in from a range of remaining manufacturers. Our results show that some of these are transnational companies. Reasons for non-participation in this group are likely to be different to smaller manufacturers, who may benefit from increased government support such as grants or tax incentives to display HSR voluntarily, or flexibility to display HSR via stickers rather than incur the cost of re-labelling.

Results which show inconsistent HSR application also suggest attainment of the 70% target will require increased voluntary display of the HSR logo on low-scoring products. Previous analysis has shown that the vast majority of products with HSR ≤ 2.0 are discretionary products not recommended by the Australian Dietary Guidelines, but which make up a large proportion of Australian diets [7,24]. Our results reflect the commercial reality that these products are unlikely to display HSR voluntarily where it does not provide marketing benefit. In effect, a voluntary HSR is operating more akin to a ‘tick’ or green light on products scoring 3.0 or above, rather than fulfilling its original objective as an overall spectrum rating of the healthiness of foods. This is a major limitation on HSR’s current utility for consumers, particularly given global movement towards types of FoPL which show product unhealthfulness [3], and emerging evidence that these appear to be more effective in promoting healthier diets, by steering consumers away from less healthy foods [25]. At the very least, this suggests that HSR’s real-world effectiveness is contingent on it providing accurate and visible information on unhealthy products for consumers. It also suggests potential for future research to explore how this HSR’s capacity to ‘steer’ consumers away from these products could be enhanced, for example by incorporating red colour into the HSR graphic of products that receive a low rating [26,27].

Uncertainty in the feasibility of the 70% target increases the importance of regular and transparent monitoring of uptake. Where progress is off-target, the implementation plan could set out steps for the government to initiate a process of legislative preparation. For example, a mandatory HSR could be instituted through amendment to the Australia New Zealand Food Standards Code [28] or development of an alternative regulatory instrument such as that used under consumer law to implement a new Country of Origin Label [29]. While the Review noted that the majority of FoPL elsewhere remain voluntary, in the time since HSR was adopted in 2014, at least eight other countries have adopted a mandatory FoPL, suggesting that this is legally, practically and politically feasible [3,30]. A mandatory HSR would also provide stronger incentive for manufacturers to reformulate to obtain a higher rating [31].

Our examination of uptake by manufacturer group highlights the leadership of grocery retailers in applying FoPL, consistent with other jurisdictions with voluntary labels including France [32] and the United Kingdom [33]. Our findings show that since 2017 [12], ALDI has rapidly increased HSR uptake (from 31.3% of products to 81.9% products) to join Coles and Woolworths as the biggest users of HSR. Consistent application of the HSR label may reflect that unlike most manufacturers, retailers are much less reliant on the success of any individual product or brand [34,35]. This leadership could be further leveraged to increase the public health impact of a voluntary HSR in the next five years, for example, if retailers elected to display HSR for all products on shelf-tags alongside price information [36]. For example, existing experiments within an IGA setting demonstrated that retailer-initiated HSR signposting of five-star products on shelf tags increased sales of these items [37].

Beyond target metrics, our results provide insights for Food Ministers finalising wider Review recommendations, including clarification of the role, membership and terms of reference for HSR’s governance committees [6]. Our results suggest peak industry organisations represented on the HSRAC were only responsible for only 28.6% of all uptake and were using HSR on less than half (45.5%) of their joint product portfolio. This limited use of the system after five years despite public statements of support for HSR [38] suggests room for the government to review the terms of ongoing industry participation on committees whose terms of reference are founded on a ‘spirit of ongoing collaboration and good faith’ [4]. Reforms could consider merit-based criteria for selecting industry representatives on implementation committees, for example based on leadership in HSR uptake. Given the tension between the industry’s commercial imperatives and HSR’s public health objectives, revising the terms of reference of industry engagement to a stakeholder to be consulted rather than an equal collaborative partner on specific tasks such as reviewing the algorithm and deciding on HSR ‘anomalies’ would also protect the scientific independence of these tasks. This approach would be consistent with recent WHO recognition of the need to safeguard against conflicts of interest in the development and implementation of FoPL [39,40].

In addition to harnessing the power of retailers and reforming governance arrangements, an ongoing implementation plan for a voluntary HSR could incorporate improved and innovative incentives for uptake. The proposal for improved Guidance for Industry on HSR could reinforce existing direction for HSR to be used consistently across product ranges, and incorporate sanctions for those manufacturers who fail to do so, for example by removing the right for them to use the HSR trademark on their higher scoring products [3]. Additional incentives could include entitlement to tax deductions for product packaging expenses, or eligibility for government subsidies (such as research and development grants) only where companies have implemented HSR on all products [41].

Our analyses benefit from the use of systematically collected directly comparable annual datasets. The FoodSwitch Monitoring Dataset is robust for time trends but is weak for absolute coverage of the overall food supply, given its reliance upon four metropolitan stores in Sydney. HSR uptake for 2014 was estimated as zero given the absence of systematic collection of HSR data at this point in time, and it is likely that there was a small number of products displaying HSR logos by the end of 2014. Where a HSR was provided by a manufacturer we used this in our analysis, but where a HSR logo was not present on the label it was necessary to generate a HSR. As FVNL content and fibre are not currently mandatory on back-of-pack nutrition information panels in Australia, missing values were therefore estimated from ingredients lists, food composition databases, and other sources. While the HSR algorithm itself is not the focus of this paper, we note that proposed Review Recommendations to improve the HSR algorithm’s alignment with Australia’s Dietary Guidelines are likely to impact the HSRs received by some products in the future.

## 5. Conclusions

These findings illustrate the limits of commercial goodwill in applying FoPL voluntarily. Ongoing implementation must pair clear uptake targets and timelines with transparent and regular monitoring and a firm pathway for making HSR mandatory if necessary to provide consumers with a genuine tool to make informed and healthier choices.

## Figures and Tables

**Table 1:** HSR uptake and mean HSR by food category among products displaying HSR logo and among products either displaying the energy icon only or not using the HSR system at all.

Category	Products Surveyed (N)	Displaying HSR/Eligible to Display HSR (n/N)	Displaying HSR (%)	Mean HSR		
				HSR Logo	No HSR/Energy Icon Only	
Bread and bakery products	2237	828/2089	39.6	2.4	2.2	*
Cereal and grain products	1827	886/1784	49.7	3.9	3.4	*
Confectionery	1259	443/1119	39.6	1.5	1.2	*
Convenience foods	1441	704/1383	50.9	3.4	3.5	
Dairy	2496	745/2382	31.3	3.1	2.7	*
Edible oils and oil emulsions	377	92/368	25.0	3.1	2.4	*
Eggs	66	9/65	13.8	4.0	4.0	
Fish and fish products	596	324/594	54.5	3.9	3.4	*
Fruit and vegetables	3064	1040/2032	51.2	4.1	3.6	*
Meat and meat products	1594	512/1168	43.8	3.1	2.4	*
Non-alcoholic beverages	2143	657/1429	46.0	4.1	2.3	*
Sauces, dressings, spreads and dips	1889	515/1832	28.1	3.4	2.4	*
Snack foods	656	204/637	32.0	2.9	2.4	*
Special foods	775	107/323	33.1	4.4	3.4	*
Sugars, honey and related products	307	52/272	19.1	1.1	1.3	
Products in excluded categories	500					
Total	21,227	7118/17,477	40.7	3.4	2.6	

* *p* < 0.05.

**Table 2:** HSR uptake and mean HSR by company among products displaying HSR logo and among products either displaying the energy icon only or not using the HSR system at all.

Company ^1^	Products Surveyed (N)	Displaying HSR/Eligible to Display HSR (n/N)	Displaying HSR (%)	Mean HSR		
				HSR Logo	No HSR/Energy Icon Only	
McCain Foods	119	116/119	97.5	3.7	3.5	
Sanitarium ^+^	82	79/82	96.3	4.3	2.7	*
Coles ^	2338	1602/1733	92.4	3.0	3.0	
Simplot ^+^	423	375/423	88.7	4.0	3.0	*
Woolworths ^	1357	958/1083	88.5	3.3	3.5	
SPC Ardmona Operations	84	69/84	82.1	3.9	2.7	*
ALDI ^	1969	1419/1733	81.9	3.2	2.4	*
Nestlé ^+^	408	242/309	78.3	3.9	1.4	*
Coca-Cola Amatil ^+^	208	148/197	75.1	-	2.0	
Kellogg’s ^+^	97	70/94	74.5	3.4	1.7	*
Mars ^+^	437	215/290	74.1	3.5	1.3	*
Campbell Arnott’s ^+^	230	158/225	70.2	2.7	1.9	*
Schweppes	137	87/131	66.4	-	1.8	
Unilever ^+^	293	166/260	63.8	3.4	2.0	*
Freedom Foods Group ^+^	128	71/122	58.2	4.4	3.8	*
Lion Dairy & Drinks ^+^	245	119/229	52.0	3.9	2.6	*
George Weston Foods ^+^	118	60/118	50.8	3.8	1.9	*
The Smith’s Snackfood Company	94	24/92	26.1	3.3	2.3	*
Heinz	307	64/261	24.5	4.0	3.0	*
Goodman Fielder ^+^	195	40/175	22.9	3.8	2.5	*
San Remo Macaroni Company	150	13/150	8.7	3.5	3.4	
Ricegrowers (SunRice) ^+^	134	11/127	8.7	3.6	3.1	*
Bega Cheese ^+^	109	3/109	2.8	4.0	2.1	*
The Market Grocer	202	4/191	2.1	4.9	3.7	
Mondelēz ^+^	295	0/291	0.0	-	1.3	
Oriental Merchant	196	0/188	0.0	-	2.1	
IGA ^	289	0/161	0.0	-	2.6	
Manassen Foods	174	0/160	0.0	-	2.9	
Parmalat	136	0/135	0.0	-	3.2	
General Mills ^+^	130	0/108	0.0	-	2.5	
Murray Goulburn Co-operative Company	91	0/91	0.0	-	3.4	
All other companies	10,052	1005/8006	12.6	4.0	2.7	*
Total	21,227	7118/17,477	40.7	3.4	2.6	

^1^ Results are listed individually for manufacturers with ≥80 HSR eligible products, ^ Grocery retailers’ private-label product range, ^+^ Australian Food and Grocery Council members, * *p* < 0.05.

**Figure A1:**
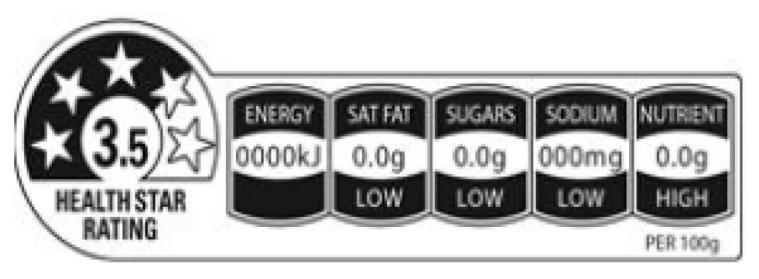
Health Star Rating + energy icon + 3 prescribed nutrient icon + 1 optional nutrient icon.

**Figure A5:**
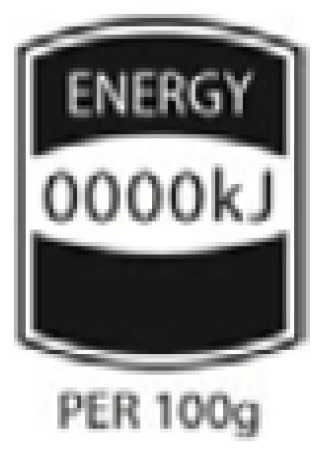
Energy icon only.

## Appendix A

Permitted variants of the Health Star Rating system graphic, extracted from the Health Star Rating Style Guide for Industry (December 2017, Version 5). Available online: http://healthstarrating.gov.au/internet/healthstarrating/publishing.nsf/Content/651EEFA223A6A659CA257DA500196046/$File/HSR%20Style%20Guide-v5.pdf.

For the purposes of our findings, we compared the mean HSR of products displaying the HSR logo (Figure A1, Figure A2, Figure A3 and Figure A4) to those carrying no HSR logo (i.e., displaying the Figure A5 energy icon only or no HSR variant at all).
